# Detection of *Klebsiella pneumoniae* in healthy poultry: Insights and perspectives from culturing and metagenomics

**DOI:** 10.1111/1758-2229.13240

**Published:** 2024-02-22

**Authors:** Håkon Kaspersen, Anne Margrete Urdahl, Hanna Karin Ilag, Fiona Valerie Franklin‐Alming, Thomas H. A. Haverkamp, Marianne Sunde

**Affiliations:** ^1^ Norwegian Veterinary Institute Ås Norway

## Abstract

Previously, *Klebsiella pneumoniae* was found to occur more frequently in healthy turkey flocks than in healthy broiler flocks in Norway. This study aimed to investigate whether this higher occurrence could be attributed to a greater abundance of *K. pneumoniae* in turkey flocks. We compared culturing, qPCR, and shotgun metagenomic sequencing for the detection and quantification of *K. pneumoniae*. Using qPCR, we found that 20.7% of broiler flock samples and 63.9% of turkey flock samples were positive for *K. pneumoniae*. Culturing revealed a significantly higher abundance of *K. pneumoniae* in turkey flocks compared to broiler flocks. However, metagenomic analysis showed no difference in the relative abundance of *Klebsiella* spp. between broiler and turkey flocks, and no correlation between the results of culturing and metagenomic quantification. Interestingly, the differential abundance of *K. quasipneumoniae* was significantly different between the two hosts. Our results indicate that Klebsiella spp. are present in both turkey and broiler flocks at relatively low levels but with a higher abundance in turkey flocks. Our findings also suggest that shotgun metagenomic studies targeting low‐abundance taxa such as *Klebsiella* have poor sensitivity when comparing groups, indicating that reliance on results from metagenomic analysis without experimental validation should be done with caution.

## Graphical abstract

This study investigates the abundance of *Klebsiella pneumoniae* in healthy poultry flocks in Norway, using culturing, qPCR and metagenomics. The results indicate a higher abundance of *K. pneumoniae* in turkey flocks compared to broiler flocks, although at an overall low level. The results also suggest that shotgun metagenomic studies targeting low abundant taxa such as *Klebsiella* have a poor sensitivity when comparing groups, indicating that care should be taken to only rely on results from metagenomic analysis without experimental validation.

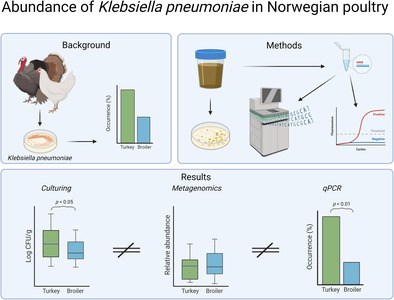

## INTRODUCTION

*Klebsiella* is a genus of gram‐negative bacteria in the *Enterobacterales* order and is found in a wide variety of ecological niches. The *Klebsiella* genus is comprised of several species, of which *K. pneumoniae* is likely to be the most studied species. *K. pneumoniae* is an important opportunistic pathogen often associated with antimicrobial resistance (AMR) and increased virulence (Wyres et al., 2020). The species is separated into several phylogroups, namely Kp1 to Kp7, and these phylogroups correspond to distinct taxa that collectively encompass the *K. pneumoniae* species complex (KpSC) (Wyres et al., 2020). These taxa include *K. variicola*, *K. quasivariicola*, *K. quasipneumoniae*, *K. africana* and *K. pneumoniae* (Wyres et al., 2020), and sub‐species thereof. Although *K. pneumoniae* is a well‐studied human pathogen, there is scarce knowledge of *K. pneumoniae* in animal reservoirs. In previous studies, we showed that *K. pneumoniae* was commonly present in the intestinal flora of turkey and broiler flocks in Norway (Franklin‐Alming et al., 2021; Kaspersen et al., 2023). *K. pneumoniae* is however not regarded as a very common cause of infection in animals. This is reflected in what is observed in Norway as well, as only sporadic clinical cases seem to be the case. Nonetheless, it is important to investigate the poultry niche as a possible reservoir of potentially pathogenic *K. pneumoniae* from a true one health perspective.

Several methods have been developed for the detection and quantification of *K. pneumoniae*, such as culturing on Simmons citrate agar with 1% inositol (SCAI) media (Van et al., 1984). Recently, a qPCR assay for the detection of KpSC phylogroups in environmental samples was published (Barbier et al., 2020). This qPCR assay utilises a 78 bp region within the *zur‐khe* intergenic region (ZKIR) as a target, which has a high specificity for KpSC phylogroups. Further, whole metagenomic sequencing (WMS) is another alternative for the detection and quantification of bacterial species or genera within a sample. To date, WMS is the most comprehensive method for the classification of the gut microbiota. WMS, qPCR and culturing of *K. pneumoniae* have previously been compared with success on human faecal samples (Lindstedt et al., 2022), but no studies have so far compared these methods for the detection and quantification of *K. pneumoniae* in samples from other hosts, such as broilers and turkeys.

By culturing, we detected a higher occurrence of *K. pneumoniae* in turkeys compared to broilers in our previous studies (Franklin‐Alming et al., 2021; Kaspersen et al., 2023). This could be attributed to a higher abundance of *K. pneumoniae* in turkeys compared to broilers. Based on these previous results, we designed the present study to quantify the abundance of *Klebsiella* in broiler and turkey ceca using culturing and metagenomic sequencing and to determine to what extent the different methods correlate. Lastly, previous culturing results (Franklin‐Alming et al., 2021; Kaspersen et al., 2023) and the ZKIR qPCR were compared with regard to the presence/absence of *K. pneumoniae* in the samples.

## EXPERIMENTAL PROCEDURES

### Laboratory analyses

#### Sampling scheme

The NORM‐VET surveillance programme for antimicrobial resistance in bacteria from feed, food and animals in Norway includes samples of the ceca of broiler and turkey flocks collected at slaughter (Norwegian Veterinary Institute, 2019). The sampling is conducted in a manner that the sampled flocks are representative of the Norwegian turkey and broiler population. From each flock, a cecum of 10 individual animals is retrieved at slaughter and sent to the laboratory within 24 h. The content of the 10 ceca is mixed into one sample, representing the respective flock. In this study, a total of 246 flock samples were included, where 174 were from broilers and 72 were from turkeys, all from 2020. All samples are listed in Data S1.

#### Bacteriological quantification

To quantify the level of *Klebsiella* in the caecal samples, 1.0 g of sample material was diluted with 9.0 mL buffered peptone water and mixed thoroughly. Then, four 1:10 dilutions were created, and 100 μL of each dilution was plated onto SCAI agar plates. Putative *Klebsiella* colonies were counted after 48 h of incubation at 37°C. Additionally, the caecal samples were plated directly onto SCAI agar without dilutions and incubated similarly as above. In both cases, Matrix‐assisted laser desorption time of flight mass spectrometry (MALDI‐TOF MS, Microflex Biotyper) was used for species confirmation of putative *Klebsiella* spp. colonies. Differences in abundance between hosts were tested with a two‐sided Wilcoxon rank test in R (R Core Team, 2021) version 4.3.0. The difference in occurrence was tested with a chi‐squared test.

#### DNA extraction

The PureLink Microbiome Purification Kit (Invitrogen) was used to extract DNA from 0.1 g of the caecal samples, with minor changes to the protocol: A 10 mM Tris‐buffer with pH 8.0 was used as an elution buffer instead of the one included in the kit. Additionally, the included homogenisation tubes were exchanged with Lysing Matrix E (MP Biomedicals) homogenisation tubes, which contain bigger ceramic beads to ensure a better homogenisation of the material. DNA concentration was determined with a Qubit assay, and DNA purity with the NanoDrop ONE.

QIAmp DNA mini kit (Qiagen) was used to extract DNA from a pure culture of CCUG 225 *K. pneumoniae* subsp. *pneumoniae*, by following the included protocol. This extract was used as a positive control for the qPCR described below.

#### ZKIR qPCR

The ZKIR‐qPCR assay was used to detect isolates from the KpSC, as previously described (Barbier et al., 2020), using the published protocol (Barbier et al., 2019). The PCR efficiency was calculated from standard curves using 7.5 ng, 750 pg, 75 pg, 7.5 pg, 750 fg, 375 fg, 45 fg and 15 fg of genomic DNA of the *K. pneumoniae* strain CCUG 225. Ct‐values, and amplification‐ and melting curves were inspected to determine if a sample was positive. Differences in the occurrence of *K. pneumoniae* between groups were tested with Chi‐squared tests in R. The sensitivity and specificity of the qPCR were calculated in relation to the culturing results.

### Shotgun metagenomic sequencing

#### Sample selection

Samples for shotgun metagenomic sequencing were selected based on the results from the culture quantification. A sample was regarded as *K. pneumoniae* positive if it was culture positive and ZKIR qPCR positive. Each positive sample was categorised into groups based on the amount of *Klebsiella* determined by the culture quantification: low (≤500 CFU/g material), medium (>500 & ≤5000 CFU/g material) and high (>5000 CFU/g material). Four samples from each group from each host species were randomly selected using the slice_sample function from dplyr (Wickham et al., 2023) version 1.1.0 to a total of 12 for each host species. Additionally, 10 *Klebsiella* negative samples (by culturing and qPCR) were randomly selected from each host species. This selection resulted in a total of 44 samples selected for sequencing. Unfortunately, due to an error in sample metadata, two broiler samples were mislabelled as turkey samples. Thus, the final number of samples for sequencing was 24 broiler samples and 20 turkey samples.

#### Sequencing

The DNA extracts of the 44 selected samples, in addition to a log‐distributed ZymoBIOMICS Microbial Community DNA standard II, were prepared by the Norwegian Sequencing Centre using the Nextera DNA FLEX (Illumina) library kit and sequenced on a NovaSeq S4 instrument, generating 150 bp paired‐end reads at an approximate depth of 40–50 million reads per sample.

### Bioinformatic analyses

#### Quality control and taxonomic classification

The Taxprofiler pipeline (Stamouli et al., 2023) version 1.1.1 was used for quality control and taxonomic classification of all shotgun reads. Briefly, reads were quality trimmed with fastp (Chen et al., 2018) version 0.23.4, followed by complexity filtering with bbduk from bbmap (Bushnell, 2019) version 39.01 and host removal with Bowtie2 (Langmead & Salzberg, 2012) version 2.4.4. Genomes for turkey (*Meleagris gallopavo*) accession number GCF_000146605.3, Broiler (*Gallus gallus*), accession number GCF_016699485.2 and the human reference genome version 19 (Bushnell, 2018) were used as references for the host removal step. Taxonomic classification was done with Kraken2 (Wood et al., 2019) version 2.1.2, using the pre‐built pluspfp database (last updated 5 June 2023). Bracken (Lu et al., 2017) version 2.7 was used to correct the read counts in the Kraken2 results, using a kmer size of 35 and a read length of 150 bp. Finally, kraken‐biom (Dabdoub, 2023) version 1.2.0 was used to generate a biom file from the Bracken reports. The biom file was imported into R (R Core Team, 2021) version 4.3.0 for further analyses using the R packages microViz (Barnett et al., 2021) version 0.10.10, phyloseq (McMurdie & Holmes, 2013) version 1.44.0, and vegan (Oksanen et al., 2019) version 2.6.4.

#### Diversity analyses

The Shannon alpha diversity was calculated at the genus level. The alpha diversity values were subjected to an analysis of variance on a linear regression model including the host species and total number of classified reads per sample. Beta diversity was calculated on the genus level by using the Bray‐Curtis distance and PCoA ordination. Statistical significance between host species was calculated with the adonis2 function from vegan, using 9999 permutations and including the total number of classified reads as a variable in the model. Dispersion of the beta diversity distances was tested with the vegan betadisper function using the spatial median method.

The top five phyla for both host species were identified by abundance, and significance between host species was tested with Wilcoxon rank tests with Bonferroni correction. A differential abundance analysis at the species level between the host species was conducted with DESeq2 (Love et al., 2014) version 1.40.2, using the raw read counts. Only species present in at least 10% of the samples, with a minimum total abundance count of 10,000 and a prevalence detection threshold count of 1000, were included in the analysis. The raw read counts were transformed using the median‐of‐ratios method implemented within DESeq2.

#### *Relative abundance of* klebsiella

A Wilcoxon rank test was used to test the difference in *Klebsiella* abundance between hosts. The relationship between the log‐transformed cfu/g from the culture quantification and the log‐transformed read counts of the *Klebsiella* genus was tested using a Pearson correlation test.

## RESULTS

### qPCR and bacteriological quantification

**FIGURE 1:**
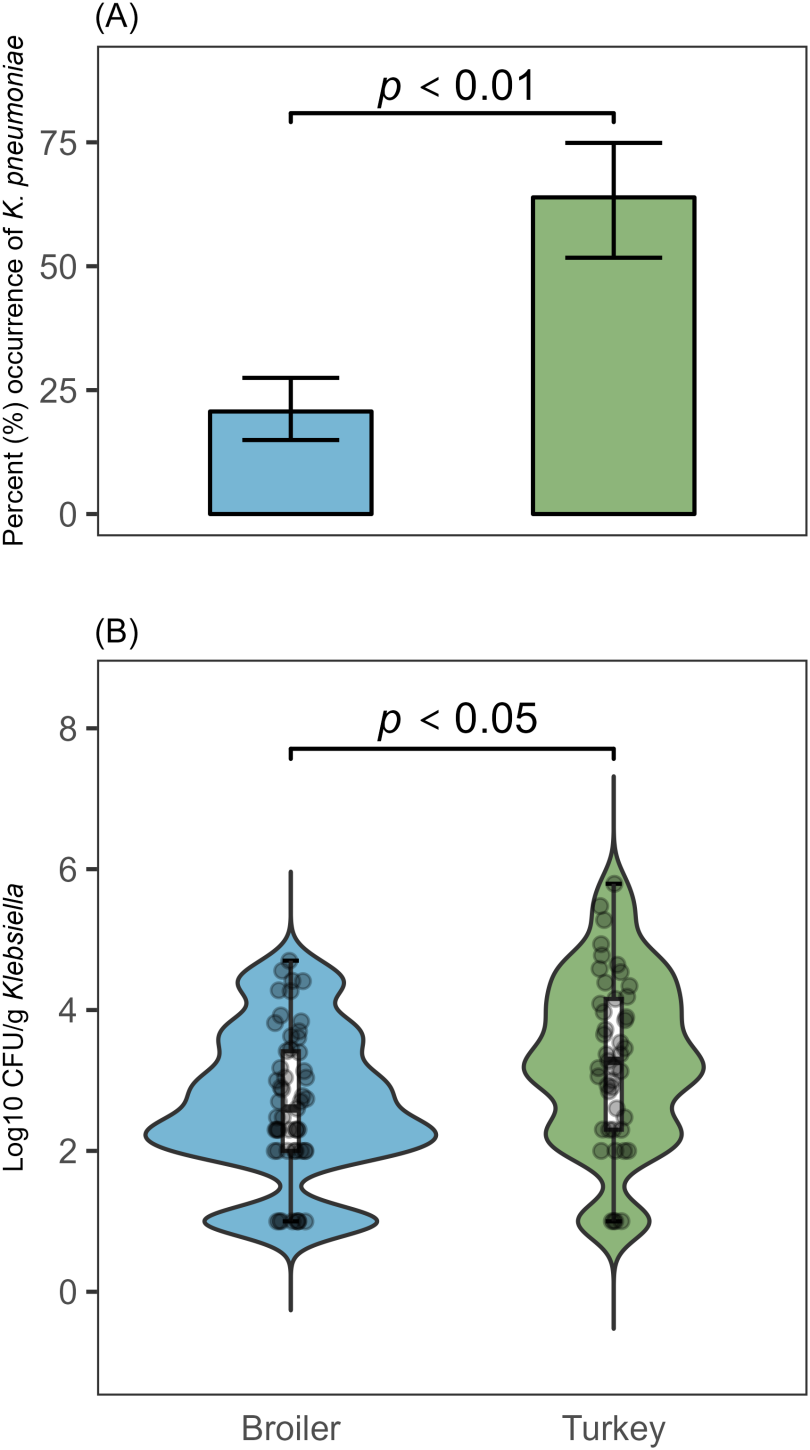
qPCR of *Klebsiella pneumoniae* and culture quantification of *Klebsiella* spp. among 246 samples from broiler and turkey flocks in 2020. (A) Calculated occurrence from qPCR results of *K. pneumoniae* among the 246 samples from broiler and turkey flocks, with 95% confidence intervals. (B) Culture quantification of *Klebsiella* spp. on SCAI medium.

Using the ZKIR qPCR assay, 36 of the 174 (20.7%) broiler flock samples and 46 of the 72 (63.9%) turkey flock samples were positive for *K. pneumoniae* (Figure 1). A significant difference in the occurrence of *K. pneumoniae* between the host species was detected using the ZKIR qPCR assay (*Χ* ^*2*^(1,N = 246) = 19.219, *p* < 0.01). The overall sensitivity and specificity of the ZKIR qPCR was calculated as 70.4% and 91.2%, respectively. The sensitivity was higher for the turkey samples (89.0%) compared to the broiler samples (54.7%), while the specificity was higher for the broiler samples (94.2%) compared to the turkey samples (77.8%).

Culture quantification revealed a median CFU/g *Klebsiella* spp. at 500 CFU/g for the broiler flock samples, and 1900 CFU/g for the turkey flock samples, excluding negative samples. A two‐sided Wilcoxon rank sum test indicated that this difference was significant (*W* = 635, *p* = 0.0128).

### Bioinformatic analyses

#### Major phyla

The most abundant phyla in the broiler and turkey samples were Bacillota (63.9% and 60.6%, respectively), followed by Bacteroidota (13.6% and 8.4%) and Actinomycetota (10.5% and 19.1%), see Table 1. The abundances of Actinomycetota (*W* = 104, *p* = 0.005) and Pseudomonadota (*W* = 354, *p* = 0.033) were significantly different between the two host species.

**TABLE 1:** Top five phyla detected in the 24 broiler and 20 turkey caecal samples from 2020.

	Wilcoxon rank test results			Relative abundance	
Phylum	Statistic	*p*‐value	Corrected *p*‐value	Broiler	Turkey
Bacillota	294	0.210	1.000	63.9	60.6
Bacteroidota	316	0.075	0.375	13.6	8.4
Actinomycetota	104	0.001	0.005	10.5	19.1
Pseudomonadota	354	0.007	0.033	5.5	4.9
Streptophyta	205	0.420	1.000	3.6	4.0

*Note*: The *p*‐values from the two‐sided Wilcoxon rank tests and the Bonferroni‐corrected *p*‐values are listed.

#### Diversity analyses

**FIGURE 2:**
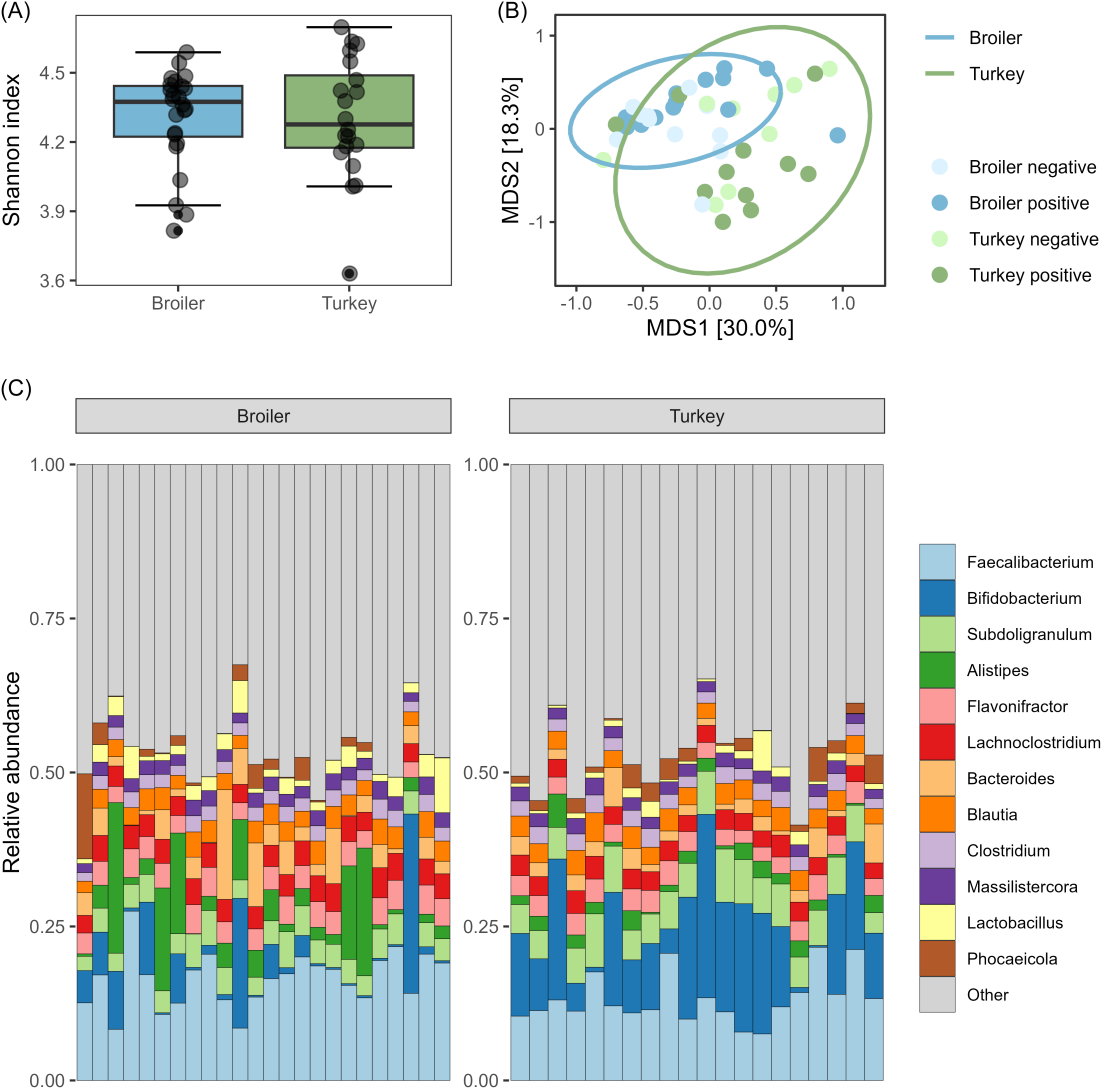
Alpha and Beta diversity and relative abundance at the genus level of the gut microbiota of 24 broiler and 20 turkey cecal samples from 2020. (A) Alpha diversity using the Shannon index. (B) PCoA ordination of Bray‐Curtis distances. The colours of the dots show if the sample was positive or negative for species within the KpSC using culturing and qPCR. (C) Relative abundance of the top 12 genera. The rest of the genera are grouped into the ‘Other’ category.

The alpha diversity was calculated using the Shannon index (Figure 2). No significant difference in alpha diversity of the gut microbiota at the genus level was detected between the host species (*p* = 0.965, Table S1). The total number of classified reads was significant in the linear model (*p* < 0.01).

Beta diversity was calculated using the dissimilarity matrix based on Bray‐Curtis distances. The Adonis test revealed a significant difference between the host species (*p* < 0.01, Table S2). The total number of reads was also significant in this model (*p* < 0.01). The dispersion test was not significant (*p* = 0.429, Table S3).

The most abundant genera in the broiler flock samples were *Faecalibacterium* and *Alistipes*, while *Faecalibacterium* and *Bifidobacterium* were the top two genera in the turkey flock samples (Figure 2).

#### Klebsiella *abundance*

**FIGURE 3:**
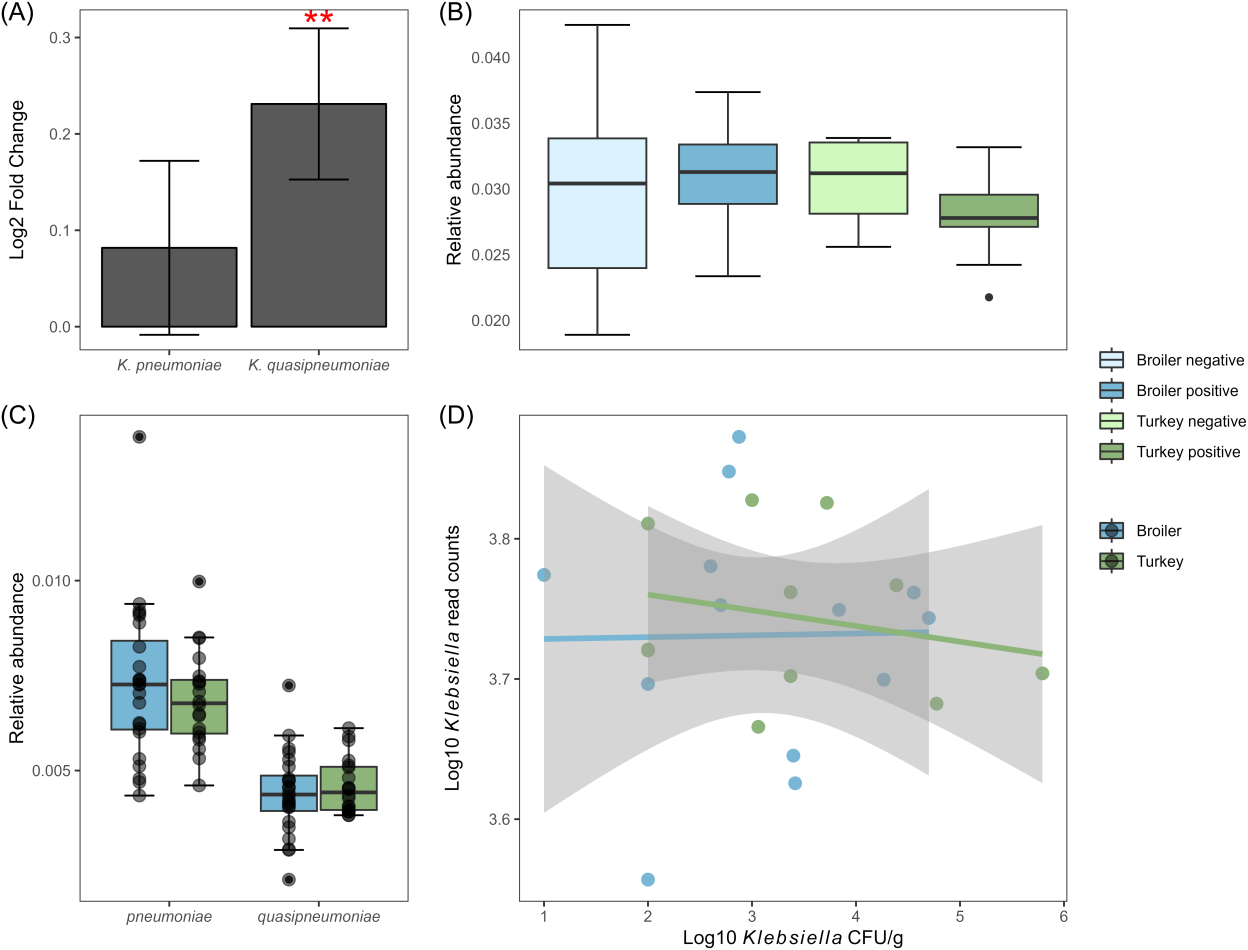
Relative abundance of *Klebsiella* spp. in 24 broiler and 20 turkey flock samples from 2020, and the correlation between culturing and shotgun metagenomic analysis. (A) Log2 fold change of *Klebsiella pneumoniae* and *K. quasipneumoniae* from the DESeq2 analysis with standard errors. Two red asterisks represent *p* < 0.01. (B) Relative abundance of *Klebsiella* spp. in each *Klebsiella* positive and negative group, calculated from the raw read counts. (C) The relative abundance of *K. pneumoniae* and *K. quasipneumoniae* in the broiler and turkey samples, calculated from the raw read counts. (D) Relationship between the logarithm of the raw read counts of *Klebsiella* spp. from Bracken and the logarithm of the colony forming units from the culture quantification.

**FIGURE 4:**
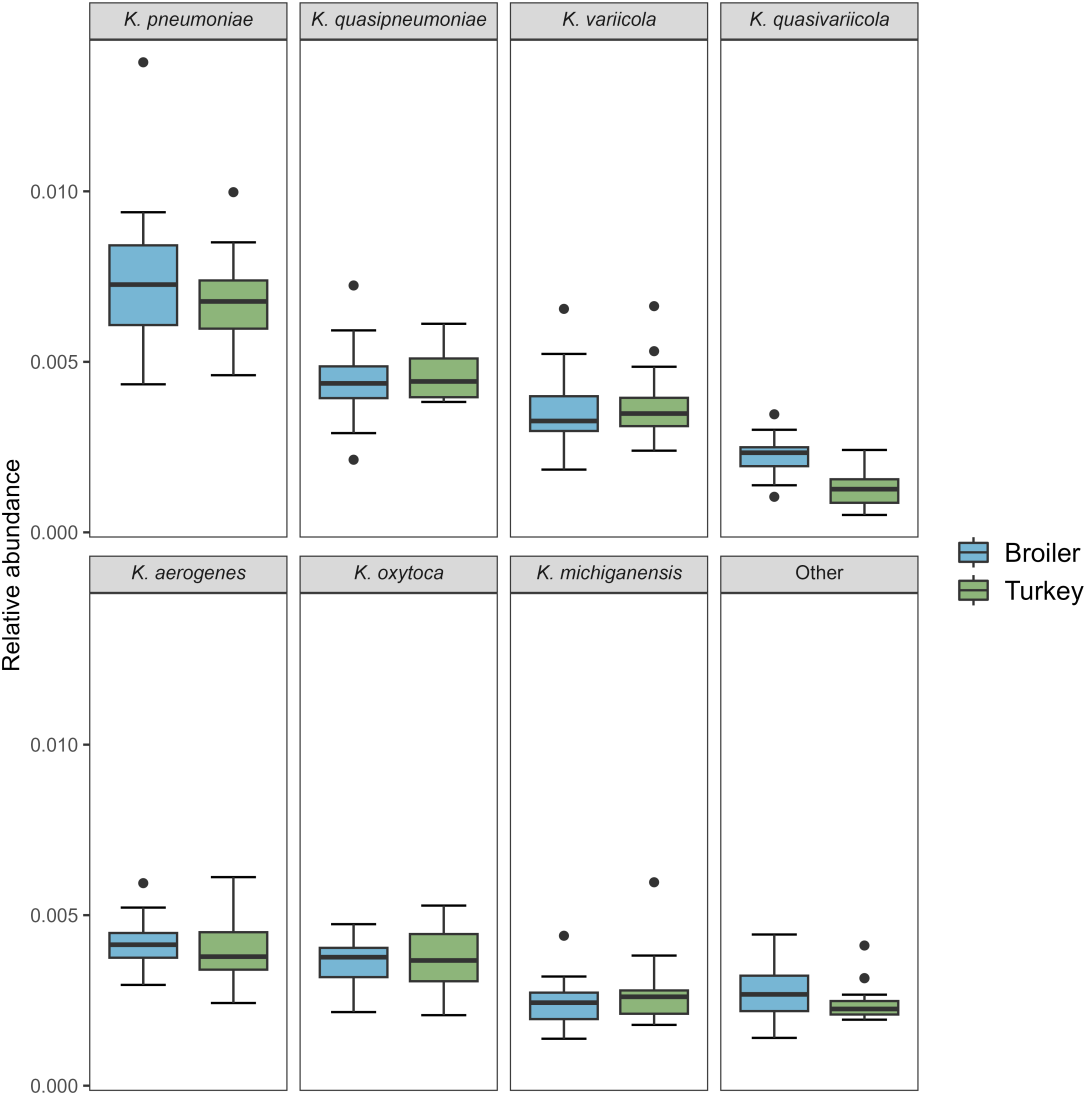
Relative abundance based on the raw read counts of the top seven classified *Klebsiella* species in 24 broiler and 20 turkey flock samples from 2020, stratified on host species. The rest of the species detected are grouped into the ‘Other’ category.

The average relative abundance for *Klebsiella* spp. was 0.030% (SD 0.006%) in the broiler samples and 0.0029% (sd 0.003%) in the turkey samples (Figure 3). No significant difference in the abundance of *Klebsiella* was detected between host species (*W* = 288, *p* = 0.2659). A weak negative correlation between the log‐transformed *Klebsiella* spp. read counts and the log‐transformed cfu/g was detected, but this correlation was not significant (*r*(22) = −0.12, *p* = 0.564). The differential abundance analysis revealed a total of 965 significant microbiota species between the host species (Data S2). Of these, *K. quasipneumoniae* was the only significant hit within the *Klebsiella* genus (*p* = 0.006). The distribution of the *Klebsiella* species between host species was relatively even, major species classified included *K. pneumoniae*, *K. quasipneumoniae*, *K. aerogenes*, *K. oxytoca*, *K. variicola*, *K. michiganensis* and *K. quasivariicola* (Figure 4).

## DISCUSSION

In this study, we quantified the abundance of *Klebsiella* spp. in caecal samples from broiler‐ and turkey flocks using culturing, and compared the results to abundance data generated with metagenomic methods. Culturing revealed a higher abundance of *Klebsiella* spp. in the Turkey samples compared to the broiler samples, but this difference was not reflected in the metagenomic data.

A higher abundance of *Klebsiella* spp. was detected in turkeys compared to broilers by using culture quantification. This corresponds well with the occurrences detected in our previous studies (Franklin‐Alming et al., 2021; Kaspersen et al., 2023), where culturing was used to detect *Klebsiella* spp. in the samples. These results are also reflected in the qPCR investigation. However, the ZKIR qPCR results revealed a relatively low sensitivity, especially for the broiler samples, while the specificity was lower for the turkey samples. The method has previously been used with success on human faecal samples (Lindstedt et al., 2022) and environmental soil samples (Barbier et al., 2020) but has not been thoroughly tested on poultry cecal samples. A total of 29 culture‐positive samples were qPCR negative, indicating a relatively high false negative rate for the qPCR. The DNA extraction process may have played a role in this, as it may not have properly captured all the potential *Klebsiella* DNA present in the culture‐positive samples. On the other hand, 13 culture‐negative samples got a positive qPCR result, indicating that the qPCR assay detected *Klebsiella* we were unable to culture. A possible explanation for qPCR‐positive and culture‐negative samples is the sample transportation time, though restricted to 24 h in most cases. Time from sampling to culturing may affect the survivability of *Klebsiella*, rendering them unable to grow on SCAI media. The median CFU/g detected in the culture‐positive and qPCR‐negative samples was 200 CFU/g, while the median for the culture‐positive and qPCR‐positive were 1350 CFU/g. Thus, the overall low abundance of *Klebsiella* in the samples may account for the low specificity and sensitivity detected.

In contrast to a study on *K. pneumoniae* in human faecal samples (Lindstedt et al., 2022), the relative abundance of *K. pneumoniae* in the metagenomes did not correlate well with the culture quantification. The combination of the potentially un‐culturable *K. pneumoniae* and the DNA extraction kit not being optimal for poultry cecal samples may have had an impact on these results. It is not likely that the sequencing depth was insufficient, as *K. pneumoniae* has previously been detected at a sequencing depth of 20 million reads/sample (Lindstedt et al., 2022). As described above, a proper DNA extraction process seems to be crucial for the detection of low‐abundant *K. pneumoniae*. Following the manufacturer's recommendations, 100 mg of each sample was extracted, and this amount of sample material may have proven to be insufficient for the proper detection of *Klebsiella* and/or *K. pneumoniae* in the metagenomic data. Also, the composition of the sample material differs significantly from human samples. This may very well have affected the DNA extraction efficiency due to the presence of possible inhibitors.

The accuracy of taxonomic classification is associated with the database and tools used. Here, we used the pluspfp prebuilt database for Kraken2, which contains sequences from known archaea, bacteria, viruses, plasmids, humans, protozoa, plants, fungi and the UniVec Core vector database. Even though the database is comprehensive, it might not represent the taxonomic diversity in poultry samples, as most sequences in such databases are derived from human samples. Utilising a larger database, such as the GenBank nt database, might have increased the number of classified reads. However, the additional classifications would likely be associated with uncultured bacteria, which would probably not provide any more insight in the current study.

Another explanation for the difference in detected abundance between the methods used in the present study could be the difference in taxonomic specificity. The SCAI medium is specific to *K. pneumoniae* and *K. oxytoca* (Van et al., 1984), and the ZKIR qPCR is KpSC‐specific (Barbier et al., 2020). The metagenomic analysis compared these to the overall presence of *Klebsiella* spp., including *K. pneumoniae*, *K. quasipneumoniae*, *K. aerogenes* and others. Despite the difference in taxonomic specificity, the largest fraction of the total *Klebsiella* spp. abundance were KpSC species. Thus, the presence of the additional non‐KpSC species is likely negligible when compared to the used KpSC‐specific methods.

The overall results on phyla composition are concordant with previous studies on both animal species (Mancabelli et al., 2016; Wei et al., 2013; Wilkinson et al., 2017). A significant difference in beta diversity was detected between the two host species. This difference was expected due to the biological difference in host species. However, the top taxa at phyla and genera levels overlap between host species, which indicates a similarity in function of the caecum in both animal species. An explanatory factor for this overlap may be the feed, as many of the same ingredients are used in feed for each of the animal species. The concept of “you are what you eat” has been investigated in several studies on human diet and gut health (Zmora et al., 2019), and also on poultry (Pan & Yu, 2014). Since diet has a large impact on the gut microbiota composition (Takeshita et al., 2021), it seems that the power‐feed regime used in both broiler and turkey industries selects for a specific microbiota in the caecum. This correlates well with findings in another study on the turkey gut microbiota (Taylor et al., 2020).

In addition to biological host species differences, several management factors may have influenced the observed diversity and taxa composition in the samples. For instance, turkeys have a longer lifespan, where the hens live for ~90 days and the roosters to around 130 days, compared to ~30 days for the broilers. In addition, very few broiler flocks (i.e., only two flocks in 2020) are treated with antibiotics, and since June 2016 they are not given coccidiostats (NORM/NORM‐VET 2022, 2023). In contrast, ~20% of all turkey flocks were treated with phenoxymethylpenicillin in 2020 (personal communication Nortura SA, Norway), and all flocks were given coccidiostat monensin. Both the treatment with phenoxymethylpenicillin and the use of monensin may have influenced the diversity estimates and major taxa in the turkey samples and thereby affected the host species comparison results. Since the present study was performed the industry in Norway has stopped using coccidiostats in turkey production as well, and a follow‐up study exploring the effect of this measure would be of interest.

Both broilers and turkeys in Norway must be considered reservoirs of *K. pneumoniae*, although with a higher occurrence in turkey as the results from our previous studies also indicated (Franklin‐Alming et al., 2021; Kaspersen et al., 2023). In the present study, we show that this is probably due to a higher abundance of *K. pneumoniae* in the turkey flock samples compared to the broiler flock samples, though seemingly in relatively low numbers, as reflected in the metagenomic analysis.

## CONCLUSIONS

Using culturing and qPCR, a higher occurrence and abundance of KpSC was detected in turkey flocks compared to broiler flocks. However, metagenomic analysis failed to detect this, as no significant difference in the abundance of *Klebsiella* spp. or KpSC was detected. This study highlights that care should be taken to only rely on results from metagenomic analysis without experimental validation.

## AUTHOR CONTRIBUTIONS

**Håkon Kaspersen:** Conceptualization (lead); data curation (equal); formal analysis (lead); investigation (equal); methodology (lead); visualization (equal); writing – original draft (lead); writing – review and editing (equal). **Anne Margrete Urdahl:** Conceptualization (lead); funding acquisition (lead); project administration (equal); supervision (lead); writing – original draft (equal); writing – review and editing (equal). **Hanna Karin Ilag:** Investigation (lead); methodology (equal); writing – review and editing (supporting). **Fiona Valerie Franklin‐Alming:** Investigation (lead); methodology (equal); writing – review and editing (supporting). **Thomas H. A. Haverkamp:** Investigation (equal); methodology (lead); supervision (equal); writing – original draft (lead); writing – review and editing (equal). **Marianne Sunde:** Conceptualization (lead); funding acquisition (lead); investigation (equal); methodology (equal); project administration (lead); supervision (lead); writing – original draft (lead); writing – review and editing (equal).

## CONFLICT OF INTEREST STATEMENT

The authors declare that they have no competing interests.

## ETHICS STATEMENT

Ethical review and approval were not required because the samples were taken after routine slaughter, under the auspices of the NORM‐VET surveillance programme.

## Supporting information


**Supplementary Data S1.** This excel file holds the sample overview of the 246 samples and individual sample accessions for the 44 sequenced samples. Below follows an explanation for the columns in the ‘Data’ sheet.

**Supplementary Data S2.** This excel file holds the results from the differential abundance analysis with DESeq2.Below follows an explanation for the columns in the ‘Data’ sheet.

**Supplementary Table S1.** Analysis of variance on the alpha diversity values. The variable origin represents the host species and the total_reads variable represents the total number of classified reads.
**Supplementary Table S2.** Adonis test of Bray‐Curtis distances. The variable origin represents the host species, and the total_reads variable represents the total number of classified reads.
**Supplementary Table S3.** Test for dispersion on the bray‐curtis distances. The test was conducted by using the betadisper function from vegan using the spatial median method.

## Data Availability

Metagenomic shotgun data has been uploaded to the SRA under the accession number PRJNA955400. See Data S1 for detailed sample accessions.
