# Supplementary material for: Detection of Klebsiella pneumoniae in healthy poultry: Insights and perspectives from culturing and metagenomics
Source: Environ Microbiol Rep. 2024 Feb 22;16(1):e13240. doi: 10.1111/1758-2229.13240 (PMC10883787; doi:10.1111/1758-2229.13240)
Supplement: Supplementary file 3 — Supplementary Table S1. Analysis of variance on the alpha diversity values. The variable origin represents the host species and the total_reads variable represents the total number of classified reads. Supplementary Table S2. Adonis test of Bray‐Curtis distances. The variable origin represents the host species, and the total_reads variable represents the total number of classified reads. Supplementary Table S3. Test for dispersion on the bray‐curtis distances. The test was conducted by using the betadisper function from vegan using the spatial median method. [file EMI4-16-e13240-s003.docx]

Supplementary Tables

Håkon Kaspersen^1*^, Anne Margrete Urdahl^1^, Hanna Karin Ilag^1^, Fiona Franklin-Alming^1^, Thomas Haverkamp^1^, Marianne Sunde^1^

^1^Norwegian Veterinary Institute, Ås, Norway

**Supplementary Table 1: Analysis of variance on the alpha diversity values**. The variable origin represents the host species, and the total_reads variable represents the total number of classified reads.

|  | Df | Sum Sq | Mean Sq | F value | Pr(>F) |
| --- | --- | --- | --- | --- | --- |
| origin | 1 | 0.00011 | 0.00011 | 0.0029 | 0.9571908 |
| total_reads | 1 | 0.70352 | 0.70352 | 18.0355 | 0.0001213 |
| Residuals | 41 | 1.59931 | 0.03901 |  |  |

**Supplementary Table 2: Adonis test of Bray-Curtis distances.** The variable origin represents the host species, and the total_reads variable represents the total number of classified reads.

|  | Df | SumOfSqs | R2 | F | Pr(>F) |
| --- | --- | --- | --- | --- | --- |
| origin | 1 | 0.21642 | 0.11889 | 7.101 | 0.0001 |
| total_reads | 1 | 0.35437 | 0.19467 | 11.627 | 0.0001 |
| Residual | 41 | 1.24956 | 0.68644 |  |  |
| Total | 43 | 1.82035 | 1.00000 |  |  |

**Supplementary Table 3: Test for dispersion on the bray-curtis distances.** The test was conducted by using the betadisper function from vegan using the spatial median method.

|  | Df | Sum sq | Mean sq | F | N. perm | Pr(>F) |
| --- | --- | --- | --- | --- | --- | --- |
| Groups | 1 | 0.002879 | 0.0028788 | 0.647 | 999 | 0.467 |
| Residuals | 42 | 0.186867 | 0.0044492 |  |  |  |
